# 表皮生长因子受体酪氨酸激酶抑制剂在晚期非小细胞肺癌一线治疗中的应用

**DOI:** 10.3779/j.issn.1009-3419.2010.01.09

**Published:** 2010-01-20

**Authors:** 阳 许, 良安 陈, 庆 田, 震 杨, 微 赵, 平 王, 星辰 刘, 春笋 李

**Affiliations:** 100853 北京，解放军总医院呼吸内科 Department of Respiratory Medicine, Chinese PLA General Hospital, Beijing 100853, China

**Keywords:** 肺肿瘤, 一线治疗, 表皮生长因子受体酪氨酸激酶抑制剂, Lung neoplasms, First-line therapy, Epidermal growth factor receptor tyrosine kinase inhibitor

## Abstract

**背景与目的:**

表皮生长因子受体酪氨酸激酶抑制剂（epidermal growth factor receptor tyrosine kinase inhibitor, EGFR-TKI）已广泛用于晚期非小细胞肺癌的二、三线治疗，但其在一线治疗中的作用尚未明确。本文旨在探讨EGFR-TKI一线治疗晚期非小细胞肺癌的疗效及安全性。

**方法:**

对77例一线使用EGFR-TKI吉非替尼或厄洛替尼治疗的晚期非小细胞肺癌患者的临床特征、治疗效果及生存时间进行回顾性分析，并对药物副作用与安全性进行评估。

**结果:**

EGFR-TKI一线治疗总有效率为33.8%，疾病控制率为68.8%。中位无进展生存时间为6.0个月，中位生存时间为8.9个月，1年生存率为61.4%。统计学分析显示：病理类型、PS评分、皮疹情况、吸烟史、EGFR突变和血清CEA变化与疗效相关；病理类型和皮疹为影响生存的独立因素。药物不良反应主要表现为皮疹和轻度腹泻。患者服用EGFR-TKI后，疾病相关症状得到缓解，生活质量明显改善。

**结论:**

EGFR-TKI一线治疗晚期非小细胞肺癌安全有效。

## Abstract (en)

**Background and objective:**

Epidermal growth factor receptor tyrosine kinase inhibitor (EGFR-TKI) has been widely used as the second- and third-line therapy in patients with advanced non-small cell lung cancer (NSCLC). However, its effect in the first-line treatment is unclear. The aim of this study was to evaluate the efficacy and safety of EGFRTKI as first-line therapy.

**Methods:**

The clinical characteristics, responses rate, disease control rate and overall survival were retrospectively analyzed in 77 chemonaive patients with advanced NSCLC. All of the patients received oral gefitinib (250 mg/d) or erlotinib (150 mg/d) until disease progression or unacceptable toxicity occurrence.

**Results:**

The overall response rate was 33.8% and the disease control rate was 68.8%. The median progression-free survival and the median survival time were 6.0 months and 8.9 months, respectively. One-year survival rate was 61.4%. Responses correlated significantly with histology, PS score, smoking history, skin rash, EGFR mutations and serum CEA. Histology and skin rash were the independent predictors of survival. Common toxicities were skin rash and mild diarrhea. EGFR-TKI could improve the clinical symptoms and the quality of life.

**Conclusion:**

EGFR-TKI is effective and well tolerated as first-line therapy in patients with advanced NSCLC.

肺癌是最常见的恶性肿瘤之一，其发病率及死亡率逐年上升。非小细胞肺癌（non-small cell lung cancer, NSCLC）约占肺癌的80%，大部分患者确诊时已为晚期。目前，含铂双药化疗仍是晚期NSCLC标准一线治疗方案，但其疗效有限。表皮生长因子受体酪氨酸激酶抑制剂（epidermal growth factor receptor tyrosine kinase inhibitor, EGFR-TKI）吉非替尼（gefitinib, Iressa）及厄洛替尼（erlotinib, Tarceva）的问世为晚期NSCLC的治疗带来了希望，现已广泛用于NSCLC的二、三线治疗，并取得了满意的效果。但其在一线治疗中的作用与地位还有待评价。本文自2005年8月以来应用EGFR-TKI一线治疗的晚期NSCLC患者77例的疗效及安全性进行回顾性分析与评价，报告如下。

## 1 资料与方法

### 1.1 一般资料

2005年8月-2009年6月在解放军总医院呼吸内科收治的经病理学确诊的晚期NSCLC患者77例，所有病人为无法耐受化疗者或不愿接受化疗者。病人在入组治疗前均签署知情同意书。77例病人中，男性28例（36.4%），女性49例（63.6%）；确诊时中位年龄64岁（21岁-88岁），年龄≥65岁者35例（45.5%），年龄 < 65岁者42例（54.5%）；病理类型为腺癌60例（77.9%），鳞癌12例（15.6%），大细胞癌3例（3.9%），腺鳞癌2例（2.6%）；吸烟者23例（29.9%），不吸烟者54例（70.1%）；PS评分0分-1分者51例（66.2%），2分-3分者26例（33.8%）；临床分期Ⅲb期22例（28.6%），Ⅳ期55例（71.4%）；77例患者有14例进行了EGFR突变检测，其中有EGFR突变者3例（21.4%），无EGFR突变者11例（78.6%）（表 1）。所有患者未接受过手术、放射治疗或化学治疗。患者自服用靶向药物起每月评估疗效，如果治疗失败，给予含铂双药化疗或最佳支持治疗。

**1:** 患者临床特征与疗效 The characteristics of the patients and the efcacy

Characteristics		*n*	RR (*n*)	χ^2^	*P*	DCR(*n*)	χ^2^	*P*
Gender	Male	28	9	0.003	0.960	17	1.351	0.245
	Female	49	17			36		
Age	≥65	35	12	0.008	0.930	21	2.333	0.127
	< 65	42	14			32		
PS score	0-1	51	18	0.158	0.691	40	6.489	0.011
	2-3	26	8			13		
Histology	Adenocarcinoma	60	26	11.122	0.001	47	11.438	0.001
	Others	17	0			6		
Stage	Ⅲb	22	11	3.629	0.057	18	2.421	0.120
	Ⅳ	55	15			35		
Smoker	Yes	23	41	3.932	0.047	11	6.745	0.009
	No	54	22			42		
Skin rash	Yes	43	23	16.937	0.000	38	17.333	0.000
	No	34	3			15		
EGFR mutation	Yes	3	3	-	0.028^*^	3	-	0.209^*^
	No	11	2			5		
	Unkown	63	-	-	-	-	-	-
Serum CEA	Decline	28	20	28.536	0.000	27	22.818	0.000
	Unchanged	22	4			16		
	Increase	27	2			10		
^*^: *Fisher* exact test. RR: response rate; DCR: disease control rate.								

### 1.2 治疗方法

吉非替尼（易瑞沙）250 mg/d或厄洛替尼（特罗凯）150 mg/d，直至肿瘤进展或因毒副反应无法耐受而停药，并根据患者情况行含铂双药化疗或最佳支持治疗。

### 1.3 疗效与毒副作用评价标准

按照最新实体肿瘤疗效评价标准（RECIST）1.1版进行疗效评估，分为完全缓解（complete response, CR）、部分缓解（partial response, PR）、疾病稳定（stable disease, SD）和疾病进展（progressive disease, PD），计算有效率（response rate, RR）和疾病控制率（disease control rate, DCR）。定期复查血常规、血生化、血清肿瘤标志物、肝肾功能、胸部CT、头颅CT、腹部B超及全身同位素骨扫描等，进行疗效判定。无进展生存时间（progression-free survival, PFS）起点为患者首次用药，终点为疾病进展；总生存期（overall survival, OS）起点为患者首次用药，终点为患者死亡或末次随访。随访时间截至2009年8月31日。根据美国国立癌症协会通用毒性标准CTC 3.0版，对毒性反应进行观测、记载和评价。

### 1.4 临床相关症状改善及生存质量评估

参照肺癌症状量表（LCSS研究量表）^[1]^，观察用药后患者在食欲、疲乏、咳嗽、呼吸困难、咯血和疼痛方面的变化，以总分上升≥25分为症状改善。

### 1.5 统计学分析

使用SPSS 13.0和CHISS统计学软件进行数据处理。疗效相关因素分析采用单因素χ^2^检验、*Fisher*精确检验和多因素*Logistic*回归法。生存分析采用*Kaplan-Meier*法、*Log-rank*检验以及*Cox*多因素回归模型。*P* < 0.05为差异有统计学意义。

## 2 结果

### 2.1 临床疗效

全组共77例患者均可以评价疗效，其中CR 4例（5.2%），PR 22例（28.6%），SD 27例（35.0%），PD 24例（31.2%）。RR为33.8%，DCR为68.8%。

### 2.2 有效率与患者临床特征的关系

单因素χ^2^检验显示，有效率与患者病理类型、吸烟史、皮疹情况、EGFR突变和血清癌胚抗原（carcinoembryonic antigen, CEA）变化高度相关（表 1）。进一步通过*Logistic*多因素回归分析显示，有皮疹、治疗后血清CEA值下降的患者一线治疗的有效率明显高于无皮疹、治疗后血清CEA值不变或上升的患者，差异具有统计学意义（表 2）。

**2:** 患者有效率及疾病控制率的*Logistic*多因素分析 *Logistic* multivariable regression analysis of response rate and disease control rate

Variable	RR				DCR		
	OR	95%CI	*P*		OR	95%CI	*P*
Gender	0.46	0.082-2.549	0.372		0.46	0.096-2.184	0.327
Age	0.25	0.057-1.136	0.073		0.49	0.163-1.476	0.205
Histology	973.40	0.060-15826.530	0.164		3.33	0.817-13.606	0.093
Stage	0.85	0.257-2.793	0.785		0.55	0.126-2.429	0.434
Smoking history	2.41	0.227-25.518	0.466		1.30	0.246-6.894	0.756
PS score	0.19	0.025-1.528	0.119		1.90	0.576-6.250	0.293
Rash	17.88	2.340-136.566	0.005		3.64	1.058-12.557	0.041
Serum CEA	3.16	1.189-8.408	0.021		1.29	0.694-2.410	0.418

### 2.3 DCR与患者临床特征的关系

单因素χ^2^检验显示，疾DCR与患者PS评分、病理类型、吸烟史、皮疹情况和血清CEA变化高度相关（表 1）。进一步通过*Logistic*多因素回归分析显示，有皮疹的患者一线治疗的DCR明显高于无皮疹的患者，差异具有统计学意义（表 2）。

### 2.4 生存情况

中位PFS为6.0（1-39.6）个月（图 1），中位OS为8.9（1.5-48.4）个月（图 2），1年生存率为61.4%。*Kaplan-Meier*生存曲线显示，病理类型和皮疹对患者的生存有显著影响（图 3A，3B）。*Log-rank*检验显示：腺癌、不吸烟、PS评分0分-1分、有皮疹、血清CEA治疗后下降患者的PFS较长（表 3）；年龄≤65岁、腺癌、不吸烟、PS评分0分-1分、有皮疹患者的OS较长（表 3）。*Cox*多因素生存分析提示，病理类型和皮疹为影响生存的独立因素（表 4）。

**1:**
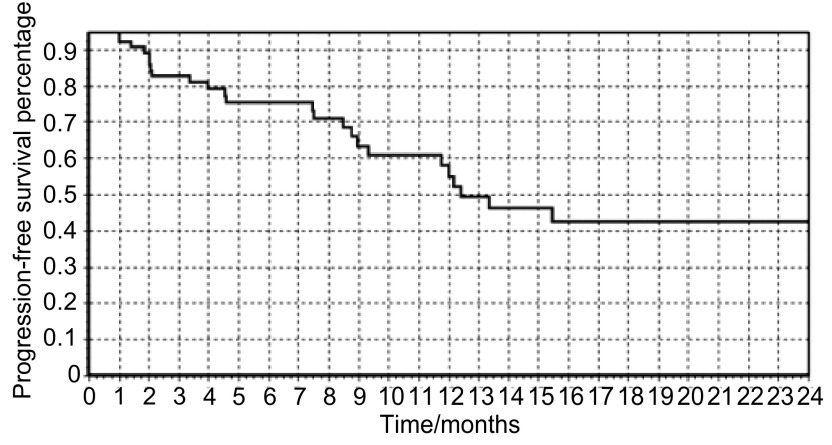
患者的*Kaplan-Meier*无疾病进展生存曲线 *Kaplan-Meier* plots of progression-free survival in all patients

**2:**
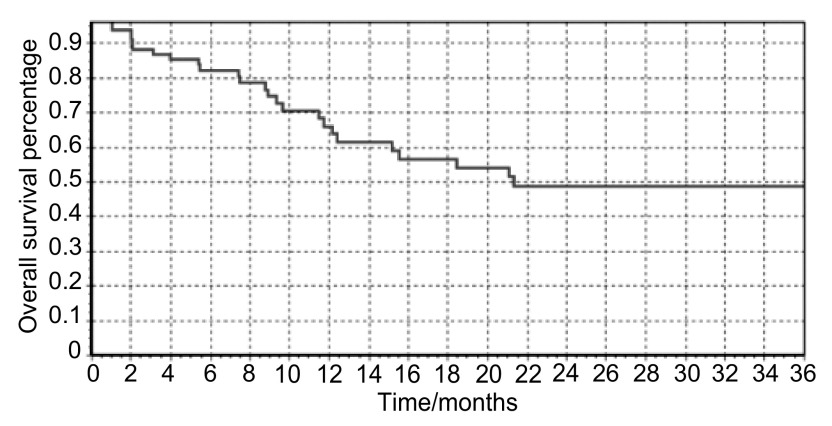
患者的*Kaplan-Meier*生存曲线 *Kaplan-Meier* plots of overall survival in all patients

**3:**
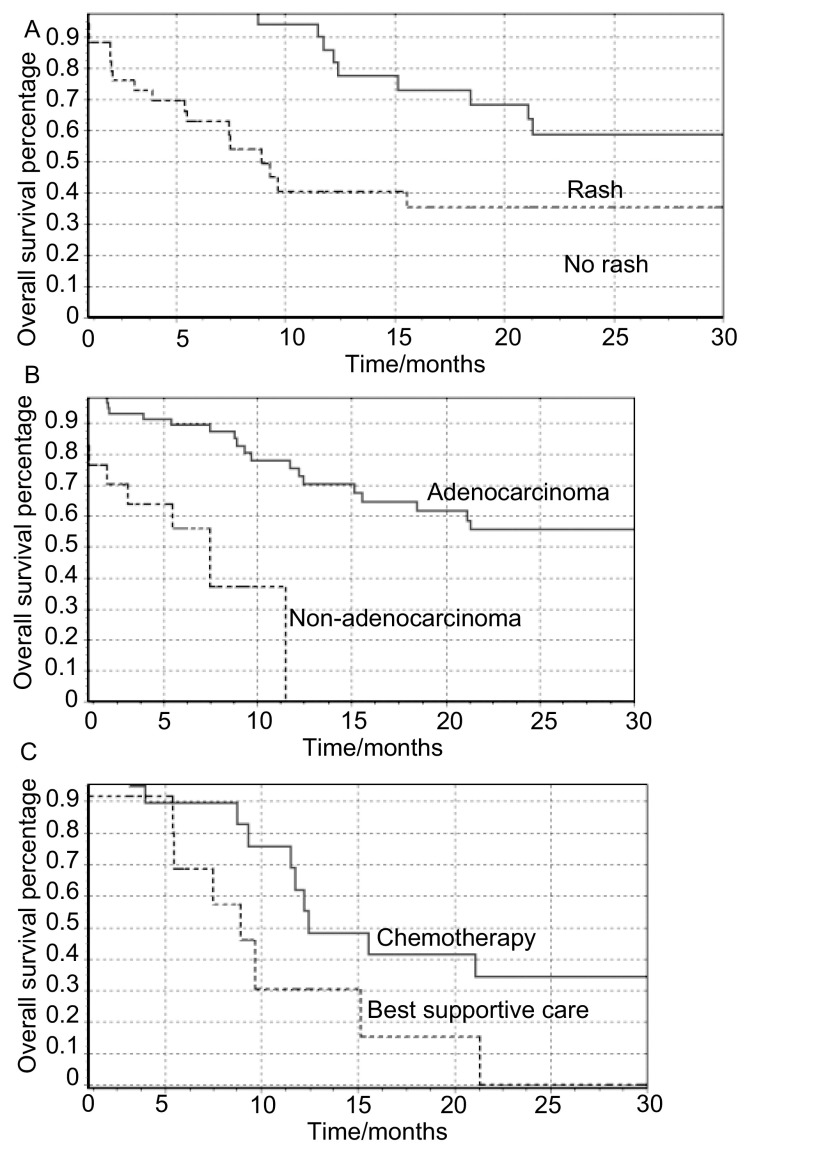
*Kaplan-Meier*生存曲线 *Kaplan-Meier* survival curves

**3:** 患者无进展生存期及总生存期的*Log-rank*检验 *Log-rank* test to comparing the survival of patients

Variable	PFS			OS	
	*Log-rank*	*P*		*Log-rank*	*P*
Gender	3.197	0.074		2.985	0.084
Age	2.149	0.143		4.259	0.039
Histology	19.863	< 0.001		16.573	< 0.001
Stage	2.300	0.129		2.784	0.095
Smoking history	9.438	0.002		8.117	0.004
PS score	9.172	0.003		9.059	0.003
Rash	15.060	0.000		10.365	0.001
Serum CEA	7.596	0.006		3.555	0.059
PFS: progression-free survival; OS: overall survival.					

**4:** 患者无进展生存期及总生存期的多变量*Cox*模型分析 *Cox* multivariate analysis of progression-free survival and overall survival

Variable	PFS				OS		
	RR	95%CI	*P*		RR	95%CI	*P*
Gender	1.43	0.412-4.986	0.571		0.95	0.273-3.306	0.936
Age	1.00	0.420-2.390	0.997		1.62	0.685-3.837	0.272
Histology	4.95	1.599-15.317	0.005		3.34	1.123-9.933	0.030
Stage	2.39	0.791-7.230	0.122		2.49	0.866-7.153	0.091
Smoking history	1.14	0.278-4.665	0.856		1.91	0.447-8.148	0.382
PS score	1.61	0.611-4.240	0.336		1.98	0.834-4.685	0.122
Rash	3.34	1.427-7.820	0.005		2.54	0.975-5.172	0.049
Serum CEA	232	0.909-5.906	0.078		1.56	0.658-3.713	0.311

### 2.5 疾病相关症状改善及生存质量评价

77例患者中有74例在治疗前存在肺癌相关症状。经EGFR-TKI治疗后，39例病人症状得到改善（总分上升≥25分），改善率为52.7%（39/74）。主要表现为咳嗽、胸痛减轻，咯血减少，胸闷气短改善，乏力缓解，食欲好转。症状缓解中位时间为10天。

### 2.6 毒副反应

临床观察与药物相关的不良反应依次为：皮疹、腹泻、恶心、疲劳、皮肤干燥、呕吐及肺间质纤维化（表 5）。未观察到肝功能异常及血液学毒性。不良反应均为1度、2度，经对症处理多可缓解。

**5:** EGFR-TKI治疗的毒副反应 Adverse events of EGFR-TKI

Adverse event	Grade 1	Grade 2	Grade 3	Grade 4	Total (%)
Rash	31	12	0	0	43 (55.8%)
Diarrhoea	19	2	0	0	21 (27.2%)
Nausea	6	2	0	0	8 (10.4%)
Fatigue	6	1	0	0	7 (9.1%)
Dry skin	2	0	0	0	2 (2.6%)
Vomiting	2	0	0	0	2 (2.6%)
ILD	2	0	0	0	2 (2.6%)
ILD: interstitial lung disease.					

### 2.7 后续治疗

截至随访时间点，有37例病人出现PD，其中18例病人不能耐受化疗而接受了最佳支持治疗（best supportive care, BSC），另外19例病人接受了化疗。19例病人中有6例病人给予多西他赛+顺铂治疗，4例病人给予多西他赛+卡铂治疗，6例病人给予培美曲塞单药治疗，给予吉西他滨+卡铂、吉西他滨+顺铂、紫杉醇+铂类治疗的病人各1例。经化疗后，6例病人由PD转为SD，分别为经培美曲塞治疗3例，多西他赛+顺铂治疗2例，多西他赛+卡铂治疗1例。其余病人仍评价为PD。接受后续治疗患者的生存曲线见图 3C。

## 3 讨论

晚期NSCLC一线治疗中，由E1594研究^[2]^确立的以铂类为基础的双药化疗方案是目前的标准治疗方案。E1594研究显示，4种第三代含铂化疗方案一线治疗Ⅲb期-Ⅳ期NSCLC患者的RR为17%-22%，中位OS为7.4个月-8.1个月，1年生存率为31%-36%。目前，含铂双药化疗的疗效已经达到平台期，在延长生存时间、减少不良反应以及提高生活质量等方面难以进一步提升，因此迫切需要寻找新的治疗模式及药物。分子靶向药物的出现让人们看到了希望。EGFR-TKI是一类较早应用于临床的分子靶向药物，它通过抑制酪氨酸激酶活性，阻断信号传导，进而抑制肿瘤细胞增殖、侵袭、转移^[3]^。INTEREST^[4]^、BR.21^[5]^、TRUST等多中心临床研究确立了EGFR-TKI类药物（吉非替尼、厄洛替尼）在晚期NSCLC二、三线治疗中的地位。但其在一线治疗中的地位还有待研究。

2005年8月开始，我们在知情同意的前提下，对77例不能耐受化疗或不愿接受化疗的晚期非小细胞肺癌患者给予EGFR-TKI一线治疗。治疗有效率为33.8%，疾病控制率为68.8%，中位无进展生存时间为6.0个月，中位生存时间为8.9个月，1年生存率为61.4%，以上结果优于E1594的一线含铂双药化疗方案。IPASS研究显示^[6]^，对于经临床选择的晚期NSCLC患者，吉非替尼一线治疗疗效好于常规化疗。本研究包含部分身体状况差、不能耐受化疗患者，但总体疗效、生存结果仍优于标准一线含铂双药化疗方案，充分显示出EGFR-TKI作为晚期NSCLC一线治疗方案切实可行。

本研究从以下几个方面分层探讨了患者临床特征和生物学标志物对疗效及疾病预后的影响。

患者基本特征与疗效及生存的关系：本研究对患者基本特征与疗效的相关性分析显示，病理类型、吸烟史与疗效及生存相关，提示腺癌、不吸烟患者可从EGFRTKI治疗中获益更多。虽然BR.21^[5]^等研究表明，女性是EGFR-TKI治疗的良好预后因素，但本实验未显示性别对生存有影响。Chang等^[7]^也指出性别与EGFR-TKI类药物吉非替尼的长期生存无关，因此性别是否对预后有影响，仍需进一步扩大病例数来求证。本实验多因素分析显示，疗效与患者年龄、PS评分、临床分期无关，提示老年、体力状况较差、临床分期较晚的患者也可通过EGFR-TKI一线治疗获益。EGFR-TKI为不能耐受化疗的患者提供了更多可选的一线治疗模式。

患者皮疹情况对疗效及生存的预测：BR. 21和TRUST研究发现，EGFR-TKI治疗中皮疹的出现是患者临床及生存获益的信号。本实验将皮疹列为一项观测指标，经统计学分析显示，与0度皮疹相比，1度和2度皮疹患者的有效率和疾病控制率显著提高，无疾病进展生存时间和总生存时间显著延长，皮疹是生存的独立预测指标，与BR.21和TRUST研究结果相符。皮疹多在用药2周内出现，因此可以根据患者皮疹情况，早期预测疗效及预后，指导下一步治疗。

患者EGFR突变对疗效及预后的影响：IPASS研究揭示EGFR突变阳性患者可从EGFR-TKI治疗中获益更多。本研究仅有14例患者检测EGFR突变，经单因素分析仍显示EGFR突变阳性患者的疗效好于EGFR突变阴性患者。多中心研究显示中国人EGFR突变率为30%^[8]^，本实验14例患者中有3例检测出EGFR突变，突变率为21.4%，低于报道水平，这与本实验检测样本量少有关。目前，肿瘤标本的不易获取已成为临床上EGFR检测的瓶颈，而从其他如血液标本检测基因突变的方法尚未达成统一共识，因此有待一种公认标准检测方法的确立。

患者血清CEA对疗效及预后的评价：临床上我们在评价EGFR-TKI药物疗效时，发现有些患者服药后临床症状有明显改善，但影像学未有相应改变。EGFR-TKI药物主要通过抑制肿瘤细胞信号传导而发挥抗肿瘤作用，能否仅凭影像学病灶体积变化来评估靶向药物的疗效还有争议。因此，有必要寻找辅助指标完善对疗效的评价。癌胚抗原CEA是血清肿瘤标志物之一，本研究统计学分析显示，服药前后患者血清CEA的变化与疗效及生存高度相关。其中治疗后血清CEA下降组疗效较好、无进展生存期较长，CEA不变组居中，CEA升高组疗效较差、生存期较短。本实验为回顾性研究，能否将血清CEA真正运用于临床疗效及预后的评价还有待前瞻性研究进一步验证。

EGFR-TKI最显著的特点之一是药物不良反应少^[9]^。本研究EGFR-TKI治疗的不良反应主要为皮疹和腹泻，均为轻中度，经对症处理多可缓解，未观察到肝功能异常及血液学毒性，患者整体耐受性较好。EGFR-TKI另一显著的特点是能提高患者生活质量。本实验发现，经EGFR-TKI治疗后，患者疾病相关症状迅速得到缓解，生活质量有明显改善。

综上所述，EGFR-TKI一线治疗可与标准一线含铂双药化疗方案相媲美，且副作用更少，耐受性更好。老年、不能耐受化疗的患者也可通过EGFR-TKI一线治疗获益，可将其作为一线方案。在EGFR基因突变等分子标志物检测的指导下，EGFR-TKI将更好的用于一线治疗，使晚期NSCLC患者获益。
